# Postmortem Interval Estimation in Saudi Arabia: Why Extreme Climate Demands a Saudi-Specific Forensic Framework

**DOI:** 10.7759/cureus.107765

**Published:** 2026-04-26

**Authors:** Abdulkreem Al-Juhani

**Affiliations:** 1 Forensic Medicine, Forensic Medicine Center, Jeddah, SAU; 2 Department of Surgery, King Abdulaziz University Faculty of Medicine, Jeddah, SAU

**Keywords:** death investigation, forensic entomology, forensic pathology, postmortem interval, saudi arabia

## Abstract

Accurate estimation of the postmortem interval (PMI) remains one of the most difficult and contested tasks in forensic medicine. In Saudi Arabia, this challenge is magnified by extreme heat, aridity, marked microenvironmental variation, and the limited availability of locally validated decomposition and entomological datasets. Traditional markers such as body cooling, rigor mortis, lividity, decomposition stage, and insect development are not inherently obsolete; rather, they become unreliable when they are applied as if they were universally transferable across climates. This editorial argues that PMI estimation in Saudi Arabia should move beyond isolated method-based interpretation toward a climate-specific, multimodal, probability-based framework. The most defensible approach is one that integrates scene thermodynamics, insect access, habitat type, local insect developmental data, and explicit uncertainty reporting. In hot arid settings, scientific caution is not a weakness; it is the basis of forensic credibility.

## Editorial

Determining the postmortem interval (PMI) remains one of the most consequential responsibilities in forensic practice. Investigative timelines, suspect alibis, and judicial reconstruction may all depend on whether the estimated time since death is scientifically defensible. Yet the literature has long made one point unmistakably clear: no single PMI method is sufficiently accurate across all postmortem stages, body conditions, and environmental settings. Traditional tools such as algor mortis, livor mortis, rigor mortis, vitreous chemistry, and gross decomposition are highly sensitive to ambient temperature, body habitus, premortem physiology, clothing, and scene-specific conditions [1,2].

This limitation becomes especially important in Saudi Arabia, where climate is not a background variable but a dominant forensic force. Extreme heat, desiccation, enclosure effects, and substantial microclimatic differences between vehicles, rooftops, shaded courtyards, desert margins, and indoor environments can shift postmortem changes in ways that make generalized PMI formulas dangerously overconfident. The central question is therefore no longer whether PMI can be estimated in Saudi Arabia, but whether it can be estimated responsibly without a framework built for Saudi conditions [1,2].

Saudi Arabia represents a particularly challenging setting for PMI estimation because extreme heat, aridity, and marked scene-to-scene microclimatic variation can distort the progression of classical postmortem changes and limit the transferability of models developed in temperate environments [1,2].

Estimating the PMI in Saudi Arabia presents a fundamentally complex challenge because environmental conditions act not merely as modifying factors, but as primary drivers of postmortem change. Classical forensic indicators - including algor mortis, rigor mortis, livor mortis, decomposition staging, and forensic entomology - are all highly sensitive to external variables. While these methods remain scientifically valid, their reliability becomes conditional when applied outside the environmental contexts in which they were originally developed and validated [1,2].

In hot arid regions such as Saudi Arabia, temperature exerts a disproportionate influence on both biochemical and physical postmortem processes. Elevated ambient temperatures accelerate enzymatic activity, bacterial proliferation, and tissue breakdown, effectively compressing decomposition timelines compared to temperate climates. However, this acceleration is not linear or uniform. Factors such as solar radiation, nocturnal cooling, airflow, and surface contact create microenvironmental variability that can produce markedly different decomposition patterns even within the same geographic location. As a result, PMI estimates derived from generalized temperature-based models may be inherently unstable in such settings [1-3].

A critical issue arises from the discordance between different PMI estimation methods under extreme conditions. Temperature-based approaches may suggest rapid postmortem progression, while morphological indicators - particularly in cases of partial desiccation - may give the impression of delayed decomposition. Simultaneously, entomological evidence may reflect either accelerated colonization due to high temperatures or delayed colonization due to restricted insect access in enclosed or shaded environments. These discrepancies are not methodological failures but reflect the coexistence of multiple biological “clocks,” each governed by distinct environmental and ecological constraints [1,2].

Forensic entomology, often considered one of the most reliable tools for estimating PMI in advanced decomposition stages, is particularly sensitive to regional ecological variation. Studies conducted in Riyadh have demonstrated significant differences in insect succession patterns between indoor and outdoor environments, with species such as Chrysomya albiceps playing a dominant role in colonization [4]. Additional research involving animal carcasses in different habitats - including urban, agricultural, and desert settings - has shown that insect community composition and succession timing can vary substantially within short spatial distances [5]. These findings underscore that insect-based PMI estimation cannot be accurately interpreted without considering local species ecology and habitat-specific dynamics.

Moreover, developmental data for forensically important species further highlight the limitations of generalized models. Experimental work in the Jazan region demonstrated that temperature significantly alters the developmental rate of Chrysomya albiceps, a key forensic species in Saudi Arabia. Even relatively small variations in temperature resulted in measurable differences in larval development timelines, directly affecting minimum PMI calculations [5]. This variability raises concerns regarding the routine application of developmental datasets derived from other geographic regions, where climatic conditions and species adaptations may differ significantly. Without locally validated developmental data, entomological estimates risk introducing systematic error despite their apparent precision [4,5].

Beyond environmental and biological variability, a broader conceptual issue affects PMI estimation in Saudi forensic practice: the implicit assumption that different methods measure the same temporal parameter. In reality, temperature-based methods estimate time since death, whereas entomological evidence often reflects the minimum time since colonization, and decomposition-based assessments reflect cumulative tissue change influenced by multiple interacting variables [3,4]. Treating these outputs as directly interchangeable can lead to misinterpretation and overconfidence in PMI conclusions. This distinction becomes especially important in complex scenes involving delayed discovery, burial, enclosure, or environmental shielding [2,3].

Given these challenges, PMI estimation in Saudi Arabia requires a shift from deterministic to probabilistic reasoning. Rather than presenting PMI as a single fixed time point, forensic practitioners should express estimates as scientifically justified ranges that explicitly account for environmental uncertainty. This approach aligns with current forensic principles emphasizing transparency, reproducibility, and the communication of limitations in expert interpretation. Furthermore, scene investigation protocols should be strengthened to systematically document critical variables, including ambient temperature, substrate conditions, sun exposure, insect access, body position, and enclosure characteristics, as these factors are integral to accurate PMI reconstruction [1-4].

The need for a region-specific forensic framework in Saudi Arabia is therefore both scientific and practical. Such a framework should integrate multidisciplinary data from forensic pathology, entomology, and environmental analysis into a unified interpretive model tailored to hot arid climates. Establishing regional decomposition datasets, expanding insect developmental studies, and incorporating climate-specific correction factors into PMI estimation models would significantly enhance accuracy and reliability. Importantly, this framework should also emphasize the standardization of reporting practices, ensuring that PMI estimates are accompanied by clear explanations of methodology, assumptions, and uncertainty.

Ultimately, the challenge of PMI estimation in Saudi Arabia is not due to a lack of available methods, but rather to the mismatch between existing methodologies and the environmental realities in which they are applied. Addressing this gap requires not only additional research but also a paradigm shift toward climate-aware forensic interpretation. Until such advancements are fully realized, PMI determinations in hot arid environments should be approached with caution, multidisciplinary integration, and explicit acknowledgment of their inherent limitations [2-5].

PMI estimation in Saudi Arabia should no longer be approached as a simple transfer of temperate-climate formulas into a desert setting. Extreme heat, desiccation, enclosure effects, and habitat-specific insect behavior make isolated methods vulnerable to error and cross-method disagreement. The strongest path forward is a Saudi-specific, multimodal framework that treats climate as a primary variable rather than a background condition. Until such a framework is routinely adopted, PMI in hot arid settings should be expressed with scientific humility, explicit uncertainty, and local ecological context.
